# Phenotypic Variability of Andersen–Tawil Syndrome Due to Allelic Mutation c.652C>T in the *KCNJ2* Gene—A New Family Case Report

**DOI:** 10.3390/biom14040507

**Published:** 2024-04-22

**Authors:** Maria Elena Onore, Esther Picillo, Paola D’Ambrosio, Salvatore Morra, Vincenzo Nigro, Luisa Politano

**Affiliations:** 1 Department of Precision Medicine, University of Campania “Luigi Vanvitelli”, 80138 Naples, Italy; mariaelena.onore@unicampania.it (M.E.O.); esther.picillo@unicampania.it (E.P.); salvatore.morra@unicampania.it (S.M.); vincenzo.nigro@unicampania.it (V.N.); 2 Cardiomyology and Medical Genetics Unit, University of Campania “Luigi Vanvitelli”, 80138 Napoli, Italy; paoladambrosiomd@gmail.com; 3 Telethon Institute of Genetics and Medicine (TIGEM), 80078 Pozzuoli, Italy

**Keywords:** *KCNJ2* gene, Andersen–Tawil syndrome, channelopathies, Kir2.1 mutations, Arg218Trp, R218W

## Abstract

Andersen–Tawil syndrome (ATS) is a multisystem channelopathy characterized by periodic paralysis, ventricular arrhythmias, prolonged QT interval, and facial dysmorphisms occurring in the first/second decade of life. High phenotypic variability and incomplete penetrance of the genes causing the disease make its diagnosis still a challenge. We describe a three-generation family with six living individuals affected by ATS. The proband is a 37-year-old woman presenting since age 16, with episodes of muscle weakness and cramps in the pre-menstrual period. The father, two brothers, one paternal uncle and one cousin also complained of cramps, muscle stiffness, and weakness. Despite normal serum potassium concentration, treatment with potassium, magnesium, and acetazolamide alleviated paralysis attacks suggesting a dyskalemic syndrome. Dysmorphic features were noted in the proband, only later. On the ECG, all but one had normal QT intervals. The affected males developed metabolic syndrome or obesity. The father had two myocardial infarctions and was implanted with an intracardiac cardioverter defibrillator (ICD). A genetic investigation by WES analysis detected the heterozygous pathogenic variant (NM_000891.2: c.652C>T, p. Arg218Trp) in the *KCNJ2* gene related to ATS, confirmed by segregation studies in all affected members. Furthermore, we performed a review of cases with the same mutation in the literature, looking for similarities and divergences with our family case.

## 1. Introduction

Channelopathies are a group of rare genetic neuromuscular disorders (NMDs) caused by dysfunction of chloride, sodium, calcium, and potassium ion channels, which are critical for muscle membrane excitability [1]. Non-dystrophic myotonias (NDM) and periodic paralyses (PP) are included in this group of genetic NMDs.

Non-dystrophic myotonias include myotonia congenita, paramyotonia congenita, and sodium channel myotonia [2]. Myotonia congenita is caused by pathogenic mutations in genes encoding chloride (*CLCN1*) and voltage-dependent muscle sodium (*SCN4A*) channels. Two forms of myotonia congenita have been described, the autosomal dominant form, better known as Thomsen disease (MIM #160800) [3] due to heterozygous pathogenic variants in *CLCN1*, and the autosomal recessive form, known as Becker disease (MIM #255700) due to bi-allelic pathogenic variants in *CLCN1* gene. Paramyotonia congenita (MIM #168300) and sodium channel myotonia (MIM #608390), both inherited as autosomal dominant traits, are caused by pathogenic mutations in the *SCN4A* gene. However, mutations in the *SCN4A* gene have also been found in periodic paralysis conditions, due to potassium electrolyte imbalance. Hypokalemic periodic paralysis (hypoPP), hyperkalemic periodic paralysis (hyperPP), and Andersen–Tawil syndrome are included in this second group of channelopathies (PP) [1], in which muscle stiffness/weakness may be the predominant clinical symptom. 

Hypokalemic periodic paralyses (hypoPPs) are autosomal dominant conditions characterized by recurrent episodes of muscle weakness and low serum potassium concentrations (hypokalemia). 

Based on specific gene mutations, hypoPPs are subdivided into two different groups: hypokalemic periodic paralysis Type 1 (MIM #170400), due to heterozygous mutations in the *CACNA1S* gene (occurring in 80% of cases) and hypokalemic periodic paralysis Type 2 (MIM #613345) due to mutations in *SCN4A* gene (occurring in 10% of cases) [1]. 

Hyperkalemic periodic paralysis (hyperPP) is characterized by high serum potassium concentrations (hyperkalemia) [4] in individuals affected and is also caused by pathogenic mutations in the *SCN4A* gene [5,6]. 

Andersen–Tawil syndrome (ATS), also defined as Andersen cardiodysrhythmic periodic paralysis, is an autosomal dominant multisystem channelopathy characterized by periodic paralysis, prolonged QT interval, ventricular arrhythmias, and facial dysmorphisms. ATS was first reported in 1971 [7] and then characterized in 1994 [8].

Mutations in the *KCNJ2* gene were first associated with Andersen–Tawil syndrome in 2001 [9] and until now more than 90 pathogenic mutations have been reported in the literature [10]. Clinically, the hypokalemic periodic paralysis picture and cardiac abnormalities, including ventricular arrhythmias, prolonged QT interval, and prominent U wave, occur in the first or second decade of life [11]. Dysmorphic features including broad forehead, hypoplastic mandible, hypotelorism, low-set ears, widely spaced eyes, fifth digit clinodactyly, syndactyly, short stature, and scoliosis are reported in 67–75% of affected patients [12]. Mild learning difficulties are also described [13].

The *KCNJ2* gene, localized on chromosome 17 (17q24.3), encodes for the inwardly rectifying potassium channel subunits Kir2.1, a channel that can assume a homo—or hetero-meric conformation [14]. Each monomer consists of two transmembrane helix segments, M1 and M2, a single ion-selective pore, and a cytoplasmic amino (NH2), and carboxyl (COOH) domains [15]. The Kir2 subfamily consists of six members (Kir2.1–Kir2.6) encoded by *KCNJ2*, *KCNJ12*, *KCNJ4*, *KCNJ14*, *KCNJ17*, and *KCNJ18* genes, respectively [16]. While Kir2.6 is primarily expressed in skeletal muscles and Kir2.4 in neurons, Kir2.1, Kir2.2, and Kir2.3 are localized in the heart, brain, and skeletal and vascular muscles [17,18].

The pathogenic mutations causing ATS are located over the entire Kir2.1 channel structure and most of them are missense variations [10]. Based on their functional implication, the pathogenic mutations have been grouped into four different categories: trafficking deficient mutations; mutations related to phosphatidylinositol 4,5-bisphosphate (PIP2)-binding defects, the main channel modulator; mutations with reported changes in channel conformation; and mutations with defects in two or more such mechanisms [10].

Advances in NGS technologies in recent years have allowed the diagnosis of a larger number of patients with ATS, highlighting the high phenotypic variability and incomplete penetrance of this channelopathy.

We reported a three-generation family with six living individuals affected by ATS and a broad spectrum of phenotypic presentations, in which genetic investigation using whole exome sequencing (WES) identified the previously reported heterozygous loss-of-function (LoF) variant in *KCNJ2* (NM_000891.2: c.652C>T, p. Arg218Trp). 

We also carried out a review of all cases with the same mutation published in the literature, looking for similarities and divergences in the phenotype compared to members of our family case.

## 2. Detailed Family Case Description

### 2.1. Clinical Evaluation of Patients

**Figure 1:**
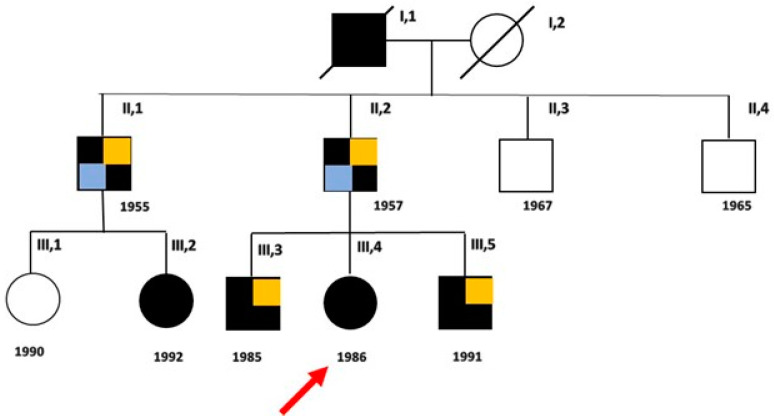
Family pedigree. Black boxes indicate the affected members and white boxes indicate unaffected individuals, after genetic test. The proband (III, 4) is indicated by the arrow. Light blue boxes indicate patients with metabolic syndrome and ischemic heart disease; orange boxes indicate patients with obesity.

A three-generation family with six living individuals affected by ATS and a broad spectrum of phenotypic presentations is described (Figure 1). 

The proband (III, 4) is a 37-year-old woman who presented episodes of muscle weakness and cramps, more common in the pre-menstrual period, since the age of 16. Creatine kinase (CK) levels were within the normal limits (160 U/L versus 190 U/L), while potassium levels were at lower reference limits (4.2 mEq/L). Treatment with potassium, magnesium, and carbonic anhydrase inhibitors alleviated the episodes of cramps and muscle weakness, suggesting a diagnosis of familial hypokalemic periodic paralysis. 

**Figure 2:**
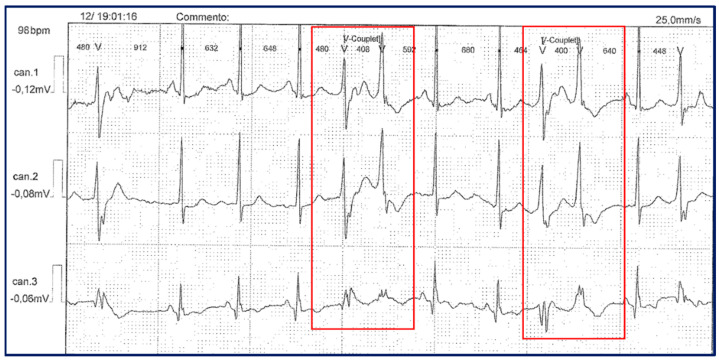
Dynamic 24 h ECG (Holter monitoring) in the proband, showing the presence of couplets of ectopic ventricular beats (red boxes).

Some years later, facial and hand dysmorphisms such as elusive chin (a mild degree of micrognathia), and then overt micro- and retro-gnathia, dental crowding, hypotelorism, high forehead, low set of ears, small hands and feet, previously overlooked, became more evident. These signs were looked for in the other members of the family and were found differently expressed in each of them, the greatest number being present in the third generation. Mild learning difficulties were observed in the proband, her father, and her younger brother. During the long follow-up (FU) (21 years), the patient independently decided to interrupt the therapy and to take only large doses of magnesium, reporting a worsening of symptoms, especially in the upper limbs with greater stiffness and weakness persisting for several days. Symptoms were exacerbated during the two pregnancies and after childbirth, partially alleviated with the re-intake of generous doses of magnesium pidolate, up to 500 mEq of Mg++. Serum potassium concentration was within the reference limits at each follow-up, but it was never possible to obtain its value during the episodes of paralysis, though she was recommended to measure it. She complained of states of anxiety and episodes of heart palpitations. Dynamic 24 h ECG, performed on several occasions during the last five years, showed the presence of ventricular and supra-ventricular ectopic beats, and couplets (Figure 2) without episodes of ventricular tachycardia.

**Figure 3:**
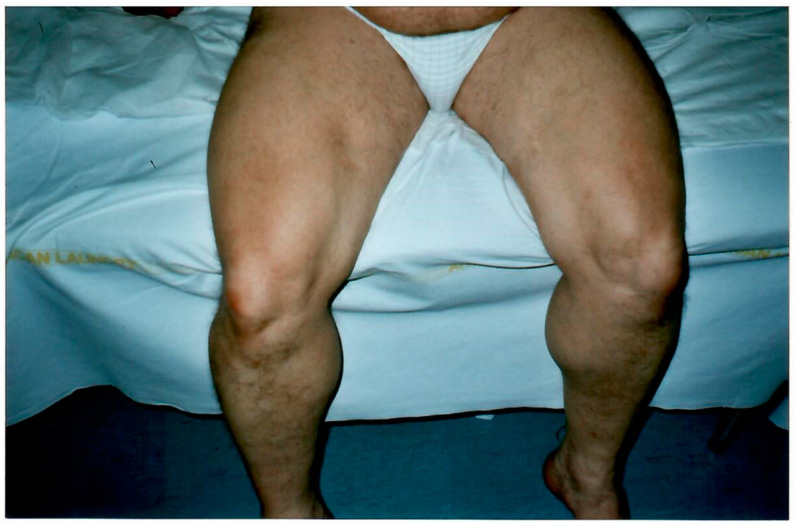
Hypertrophic appearance of lower limb muscles in patient II, 2.

Her father (II, 2), despite the presence of hypertrophic muscle masses resembling Thomsen disease, complained of cramps, contractures with myotonia, and myalgia post-exercise at femoral quadriceps (Figure 3) and calve muscles, since the age of 6. At the first consultation, he reported that his father had the same physical structure and suffered, in turn, from cramps and episodes of paralysis, and that he himself in his youth had been diagnosed with limb–girdle muscular dystrophy in a neurology department.

At clinical examination, he reported stable dysphonia, difficulties in walking and climbing stairs, and episodes of stiffness and adynamia more frequent than in the past, when they were limited to twice a year. CK levels were slightly increased (223 U/L versus 190 U/L), while serum potassium levels were at the upper reference limits. Over the years, he developed a metabolic syndrome characterized by obesity, hypertension, dyslipidemia, and type 2 diabetes. During the follow-up, he had two episodes of acute myocardial infarction (AMI), and due to the presence of ectopic ventricular arrhythmia with thousands of couplets and many dozens of runs, was implanted with an intracardiac cardioverter defibrillator (ICD) at the age of 52. To date, he presents post-ischemic dilated cardiomyopathy (ejection fraction, EF 30%) and chronic heart failure, complicated by nocturnal episodes of obstructive sleep apnea (OSA). 

The proband’s brothers (III, 3; III, 5) also have hypertrophic muscle masses and reported episodes of stiffness (twice a month), cramps, myalgias, and inconstant paresthesia in the fingertips of both hands. Patient (III, 5) sometimes complained of difficulty in muscle relaxation after prolonged contraction (myotonia). Both had increased CK levels (1157 U/L and CK 235 U/L, respectively), while serum potassium concentration ranged from normal to upper reference limits. Dysmorphic features including mandibular hypoplasia, dental crowding, clinodactyly of fifth toe, and syndactyly of II/III fingers of hands were present in both brothers, while scoliosis, hypertelorism, small ears, and short neck were present only in the youngest. Treatment with acetazolamide relieved symptoms. 

The older paternal uncle (II, 1) also presented periodic paralysis, myalgias at the posterior compartment of the thighs and calves, memory and attention disturbances, and metabolic syndrome including hypertension, diabetes, and obesity. At the age of 60, he underwent two coronary artery bypass grafts due to ischemic heart disease. 

His younger daughter (III, 2) came to our observation at the age of 27, complaining for about three years of symptoms characterized by nocturnal muscle cramps and muscle weakness upon awakening, and the presence of myotonia in the hands which appeared at the age of 6–7 years, and attenuated over times. CK values were within the normal limits, while the potassium concentration was below the normal lower limit (3.9 mEq/L). 

However, none of the patients except the proband’s father showed prolonged QT interval or severe arrhythmias during the long follow-up. None presented a prominent U wave. 

### 2.2. Genetic Analysis

**Figure 4:**
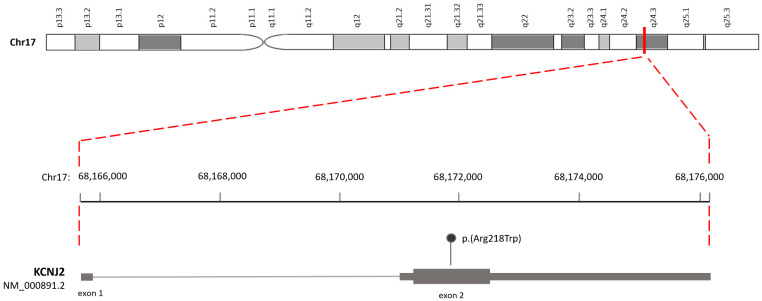
Schematic representation of the human *KCNJ2* genomic structure and position of the variant (NM_000891.2: c.652C>T, p. Arg218Trp) on exon 2.

A first genetic investigation, carried out by a targeted panel including the two main genes associated with congenital myotonias, *CLCN1* and *SCN4A* genes, was negative. However, by extending the molecular investigation to further genes associated with the described phenotype, the heterozygous missense variant NM_000891.2: c.652C>T, p. Arg218Trp in exon 2 of the *KCNJ2* gene (Figure 4) was identified. 

This variant, previously identified in the proband in an external laboratory, was validated using Sanger sequencing in our laboratory as indicated below. 

In brief, after PCR amplification of the exon 2(KCNJ2_ex2_Arg218Trp_Forward:5′-TGCTTTCATCATTGGCGCAG-3′; KCNJ2_ex2_Arg218Trp_Reverse: 5′-TCCACCATGCCTTCCAGTATG-3′). Sanger sequencing was performed using the BigDye™ Terminator v1.1 Cycle Sequencing kit (Applied Biosystems™, Foster City, CA, USA) and analyzed on a 3500 xl Genetic Analyzer (Applied Biosystems for Sanger). Automatic identification of variations was achieved by analyzing sequencing data (ABI file) using Mutation Surveyor version 3.24 software (SoftGenetics, State College, PA, USA).

**Figure 5:**
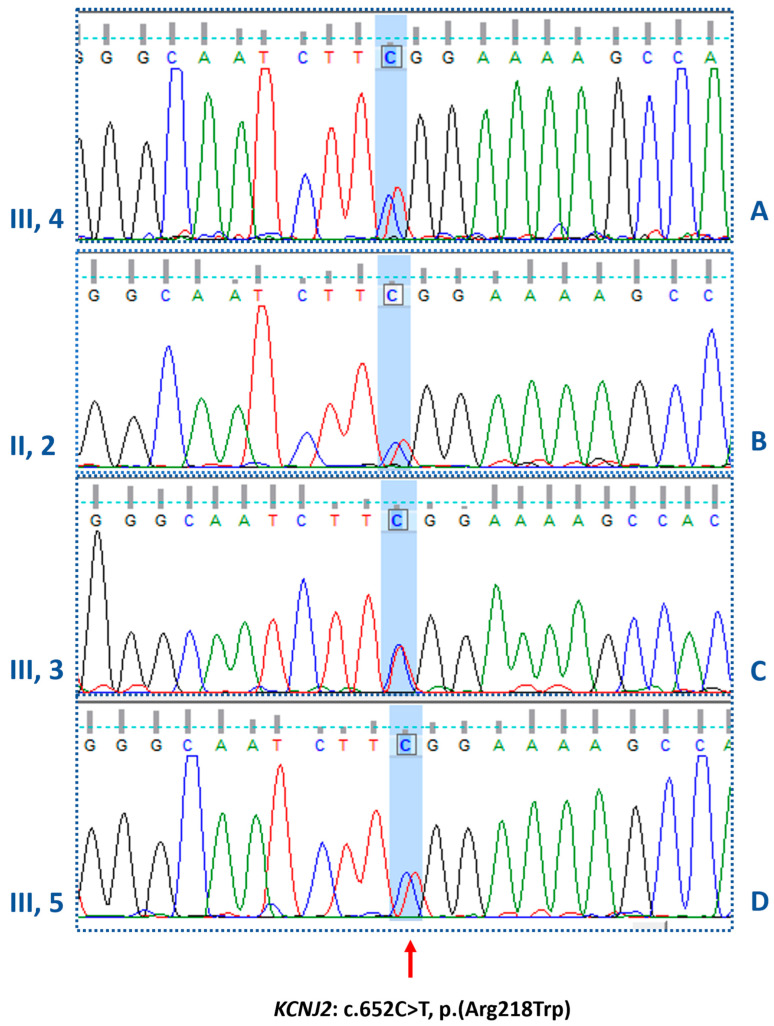
Electropherograms of the *KCNJ2* gene. In panels A, B, C, and D, the electropherograms confirm the heterozygous missense mutation (c.652C>T) identified in the proband, her father and the two brothers, respectively.

The variant, already reported in the public database ClinVar (VCV000008919.23), is predicted to be pathogenic (PP5 very strong, PM5 strong, PP3 strong, PM1 moderate, PM2 supporting), both using the default settings of Varsome (January 2024), and according to the ACMG/AMP guidelines. The analysis of segregation carried out in our laboratory, confirmed the heterozygosity in the proband, her brothers, and her father (Figure 5), and in the paternal uncle (II, 1) and the younger of his daughters (III, 2), while excluded it in the other two uncles (II, 3 and II, 4) and in the cousin (III, 1). 

## 3. Discussion

To date, nine families (not counting the one reported here) with 31 affected individuals, and 20 isolated cases carrying the heterozygous variant c.652C>T, p.Arg218Trp in *KCNJ2* gene have been described in the literature (Table 1a,b) [13,19,20,21,22,23,24,25,26,27,28,29,30,31,32,33,34,35,36].

All subjects have at least one feature of the triad commonly associated with ATS. Muscle phenotype, including muscle weakness, muscle pain, cramps, and episodes of periodic paralysis, is reported in 30/46 (65.2%) individuals. Potassium concentration is available for only five patients (9.8%), three boys and two girls, who present values ranging from 2 to 3.9 mEq/L. CK values are available for five patients (9.8%), three boys and two girls, and range from normal values to about five times the upper reference limit.

Cardiac manifestations including ventricular tachycardia (VT), premature ventricular contractions (PVC), prolonged QT interval, bidirectional or polymorphic ventricular tachycardia (bVT or pVT), syncopal episodes or cardiac arrest are reported in 25/46 (54.3%) subjects. QTc values are available for 11/31 (35.4%) male and 10/25 (40%) female patients. Prolonged QTc above the normal upper limit (440 msec in males and 460 msec in females) is present in 27.2% of male and 30% of female patients. Dilated cardiomyopathy or a high risk of cardiac sudden death are described by Schoonderwoerd [21] and Barron-Diaz [27], respectively.

Dysmorphic features are present in 32/51 (62.7%) cases. In particular, short stature or scoliosis are the most represented skeletal anomalies (21.5% and 7.8%, respectively), followed by clinodactyly (23.5%) and syndactyly (15.7%). Abnormal dentition and dental enamel discoloration are reported in three patients (5.9%) [13,27].

Some aspects found in members of our family case such as the prevalence of the disease in males (4/6, 66.7%), the great intra-familial variability concerning the age of onset and clinical presentation, the muscle phenotype as the prevalent presenting symptom, and the variable presence of dysmorphic features agree with the data present in the literature.

Age of onset varies from 6 to 34 years and muscle symptoms including cramps, post-exercise myalgia, and episodes of muscle weakness up to paralysis are the predominant features. Dysmorphic features such as elusive chin, overt micrognathia, dental crowding, hypertelorism, low set of ears, small hands and feet, clinodactyly and syndactyly of the fingers are variably present only in the third generation. 

By contrast, other aspects such as normal or above normal height, serum potassium concentration within the reference limits, concomitance of paralysis attacks with the pre-menstrual period in women, high occurrence of metabolic syndrome and ischemic heart disease in male patients, presence of myotonia in some members of the family (father and younger brother, cousin) are atypical features of ATS. 

Serum potassium concentration, recorded during the follow-up period, outside the episodes of paralysis, varies from 3.9 to 5.4 mEq/L (Table 1a,b). However, a possible correlation with potassium imbalance was supposedly based on the beneficial effect of acetazolamide on episodes of paralysis. 

Adynamia associated with the pre-menstrual period was already reported by Sarova-Pinhas et al. in 1981. They described a 17-year-old girl who suffered from episodes of flaccid paralysis during each menstrual cycle [37]. Symptoms were responsive to treatment with acetazolamide, which is a drug that acts at the nephron’s proximal convoluted tubule level by inhibiting carbonic anhydrase, an enzyme involved in maintaining the body’s acid–base balance. Through the inhibition of carbonic anhydrase activity, acetazolamide reduces the reabsorption of bicarbonate and sodium, increasing their urinary excretion, and influencing the movement of potassium from extracellular to intracellular compartments [38]. A positive response to treatment with acetazolamide or antiarrhythmic drugs blocking sodium channels, such as flecainide and mexiletine, is also reported by other authors [23,35,39,40,41,42].

Cardiac evaluation, periodically performed during the long years of follow-up—21 for the proband and her family and 15 for the uncle—showed the presence of sinus bradycardia in younger patients and the onset of ectopic ventricular beats often in pairs in the proband. Cardiovascular disease evolving to post-ischemic dilated cardiomyopathy and chronic heart failure, associated with the onset of ventricular arrhythmias requiring ICD implantation prevailed in older patients over the muscle condition. The co-existing metabolic syndrome with hypertension, obesity, type 2 diabetes, and dyslipidemia likely contributed to the rapid evolution of heart disease. 

Episodic flaccid muscle weakness (periodic paralysis), cardiac abnormalities (ventricular arrhythmias, prolonged QT interval, prominent U wave), and dysmorphic features (broad forehead, hypoplastic mandible, hypertelorism, low-set ears, widely spaced eyes, fifth digit clinodactyly, syndactyly, short stature, and scoliosis) are regarded as the triad of features typically associated with Andersen–Tawil syndrome. High phenotypic variability and incomplete penetrance of the gene causing the disease define this condition. Muscle weakness is the most immediate manifestation of the disease related to an elevated (>5.6 mEq/L), normal, or most commonly reduced (<3.5 mmol/U) serum potassium concentration. Pathogenic mutations in the *KCNJ2* gene, encoding for the inwardly rectifying potassium channel subunits Kir2.1, have been associated with ATS [43].

Here we reported a three-generation family with six living members affected by ATS, in which the genetic investigation identified the previously reported heterozygous loss-of-function (LoF) variant c.652C>T, p. Arg218Trp in the *KCNJ2* gene. The variant falls in the carboxyl (COOH) domain of the channel [9]. The residue Arg218, highly conserved, is involved in the binding of phosphatidylinositol 4,5-bisphosphate (PIP2), the main modulator of Kir2.1 channels [19]. Kir2.1 has three C-terminal PIP2 binding regions (aa 175–206, aa 207–246, aa 324–365) [43] and the substitution of residue 218 has been associated with decreased PIP2 binding and inhibition of channel function [44]. Affected patients present the clinical features characteristic of ATS including ventricular arrhythmias, dysmorphic features, and episodes of muscle weakness [22,32]. Systolic dysfunction has also been reported in these patients in the setting of dilated cardiomyopathy [21]. However, these features are not always present at the same time and can occur during the course of the disease.

The high inter- and intra-familial phenotypic variability observed in individuals with ATS [24,26,31] and this report suggests that many cases may still be undiagnosed due to the scarcity or absence of typical symptoms. The case of a man with a 60-year history of episodic weakness in the proximal limb muscles, diagnosed as ATS only at age 66, is illustrative [45]. Affected individuals can sometimes present a picture of fixed myopathy that can lead to a wrong diagnosis of muscular dystrophy [32]. 

Complicating the diagnosis is the fact that episodes of muscle weakness and periodic paralysis are not peculiar to channelopathies, but they can also be found in different conditions such as mitochondrial cytopathies, which should be taken into account in the differential diagnosis. 

Mitochondrial cytopathies represent a heterogeneous group of multisystem disorders, which preferentially affect muscle and nervous systems. They can be caused either by mutations in the maternally inherited mitochondrial genome or by nuclear DNA mutations [46,47]. Mitochondrial cytopathies are generally not considered in the diagnostic workup of patients with electrolyte disorders, which are commonly reported in mitochondrial cytopathies, often as presenting symptoms. Viering et al. [48], by studying 362 patients affected by mitochondrial diseases, found that about 80% of them had an electrolyte disorder, which was the presenting or main symptom in 13% of patients. They emphasized that mitochondrial diseases should be considered in the evaluation of unexplained electrolyte disorders. 

Owing to the non-uniform distribution of mitochondria in tissues and the co-existence of mutated and wild-type mtDNA (heteroplasmy) in these organelles, these disorders may present with a huge variety of symptoms, including muscle weakness and cardiomyopathy [46,47]. The progressive increase in mtDNA heteroplasmy causes progressive mitochondrial dysfunction leading to a loss in their bioenergetic capacity, disruption in the balance of mitochondrial fusion and fission events, and decreased mitophagy. This failure in mitochondrial physiology leads to the accumulation of depolarized and ROS-generating mitochondria. Thus, besides attenuated ATP production, dysfunctional mitochondria interfere with proper cellular metabolism and signaling pathways in cardiac cells, contributing to the development of ischemic heart disease, cardiomyopathy, and atherosclerotic vascular disease [49]. Mitochondria are known to regulate apoptotic and autophagic pathways that have been shown to play an important role in the development of cardiomyopathy and atherosclerosis [50].

In our family case, the classic mitochondrial pattern of inheritance is not conceivable, since the proband’s paternal grandfather is the first to present symptoms, with a male-to-male transmission. However, the co-occurrence of a second mutation in nuclear mtDNA genes cannot be ruled out. 

## 4. Conclusions

The clinical features typically associated with Andersen–Tawil syndrome, such as episodes of muscle weakness, cardiac arrhythmias, and dysmorphic features would require a multidisciplinary approach. 

In our opinion, closer collaboration between neuro-myologists, cardiologists, orthopedists, and geneticists in the case of patients with signs and symptoms of muscular, cardiac, or skeletal involvement described above, which, if not present at the same time, must be carefully looked for, would facilitate the clinical and genetic diagnosis of ATS.

## Figures and Tables

**Table 1:** Patients with the heterozygous variant c.652C>T, p. Arg218Trp in *KCNJ2* gene so far reported in the literature.

(a)											
ATS Familial Cases											
		Clinical Presentation			Laboratory Investigations		Cardiological Features		Dysmorphisms/Other Features		
Authors [Reference]	Relationship	Age at Onset/Sex	First Symptoms	Muscle Involvement	K^+^ Levels in mEq/L (n.v. 4.2–5.6)	CK Levels in U/L (n.v. < 90)	ECG and Echo Features at 1st Visit and During FU	QTc in msec	Facial and Other Dysmorphisms	Additional Features	Age at Last FU
Davies et al. [13]	Proband	9/F	VA	PP	n.r.	120	VA	n.r.	Abnormal dentition, dental enamel discoloration	Mild Ataxia	n.r.
	Brother	16/M	PP	PP	Low	n.r.	--	n.r.	--	--	n.r.
	Brother	14/F	PP	PP	Low	154	VA	n.r.	--	--	n.r.
	Mother	8/F	VA	PP	n.r.	n.r.	VA	n.r.	--	--	n.r.
Davies et al. [13]	Proband	24/M	Painful PP	PP	n.r.	n.r.	--	n.r.	--	--	n.r.
	Son	14/M	PP	PP	n.r.	n.r.	--	n.r.	--	--	n.r.
	Son	13/M	PP	PP	low	184	--	n.r.	--	--	n.r.
Donaldson et al. [19]	Proband	10/F	Weakness of the lower limbs; paralysis	PP; muscle weakness of lower limbs	**2.0**	n.r.	bVT; PVC; prominent U wave; LQTS; EVB	445	Micrognathia, scoliosis, clinodactyly, small ears, hypertelorism, short and wide neck, fingers syndactyly	---	29
Pouget et al. [20]	Father	38/M	Muscle pain; EVB		n.r.	n.r.	EVB	n.r.	---	---	53
Schoonderwoerd et al. [21]	Proband	18/F	Cardiac arrest	Exercise intolerance	**2.6**	n.r.	Prominent U wave; VF; DCM frequent PVCs; VT	n.r.	Mild facial asymmetry, clinodactyly, syndactyly of second and third toes	--	n.r.
	Mother	34/F	Unexpected syncopal event	--	n.r.	n.r.	Frequent PVCs; VT; DCM	n.r.	Asymmetric face, prognathia, camptodactyly of toes, syndactyly of the second and third toes	--	n.r.
	Brother	16/M	Muscle weakness	Muscle weakness of limbs	n.r.	n.r.	Prominent U wave; PVC; pVT	n.r.	Low set ears, prominent forehead, bifid uvula, webbed neck, small mandible, clinodactyly, syndactyly of the second and third toes	--	n.r.
Tengan et al. [22]	Proband	13/M	Muscle weakness	PP, weakness of proximal limb muscles	Normal	n.r.	Prolonged QT; isolated EVB	**490**	Short stature, micrognathia, retrognathia, clinodactyly of fourth and fifth fingers, arched palate, thoracic scoliosis	Obesity; OSA, daytime sleepiness	n.r.
	Daughter	6/F	Muscle weakness	Episodes of weakness	Normal	n.r.	--	n.r.	Short stature, micrognathia, clinodactyly of fourth and fifth fingers	OSA	n.r.
Bokenkamp et al. [23]	Proband	3/F	Arrhythmia	--	--	n.r.	Polymorphic EVB, prolonged QT, prominent U wave, mild aortic root dilation	**480**	Mild dysmorphic features, including broad forehead, hypertelorism, small mandible and clinodactyly	--	6
	Father	/M	Polymorphic EVB	--	--	n.r.	Polymorphic EVB	--	Mild dysmorphic features		
	Brother	6/M	Polymorphic EVB; syncope	1 episode of muscle weakness	--	n.r.	Polymorphic EVB	--	Mild dysmorphic features	--	7
Haruna et al. [24]	Proband	6/F	Syncopal events	PP	n.r.	n.r.	PVC, bVT, monomorphic VT	**483**	--	--	n.r.
	Father	38/M	Muscle weakness	PP	n.r.	n.r.	PVC	384	--	--	n.r.
	Grandfather	/M	Muscle weakness	PP	n.r.	n.r.	--	410	--	--	n.r.
Haruna et al. [24]	Proband	11/F	Muscle weakness	--	n.r.	n.r.	bVT, mVT	365	Dysmorphic features	--	n.r.
	Father	47/M	Muscle weakness	--	n.r.	n.r.	--	394	--	--	n.r.
	Brother	5/M	Myalgia	--	n.r.	n.r.	--	342	--	--	n.r.
Janson et al. [25]	Proband	10/M	VEB, bVT	PP, episodes of muscle weakness	n.r.	n.r.	EVB, bVT	--	Micrognathia, wide-spaced eyes, clinodactyly of the fifth digit	--	15
	Mother	F	PP, VT	PP	n.r.	n.r.	VT, CM	--	Micrognathia, wide-spaced eyes, clinodactyly of the fifth digit	--	n.r.
Ardissone et al. [26]	Proband	1.4/M	Acute muscle pain	Muscle pain, cramps, generalized weakness	**3.5**	**346**	Sinus Tachycardia	Normal	Broad forehead, hypoplastic mandible, low-set ears, short stature, low weight	--	4
	Mother	20/F	Muscle pain post-exercise, muscle weakness	Severe generalized muscle weakness	--	n.r.	--	--	Broad forehead, hypoplastic mandible, low-set ears, short stature, low weight	--	n.r.
	Brother	M	VT, long QT	--	--	n.r.	VT, long QT, arrhythmias	--	Broad forehead, hypoplastic jaw, low-set ears	--	n.r
	Brother	M	VT, long QT	Muscle stiffness	--	n.r.	VT, long QT, arrhythmias	--	Broad forehead, hypoplastic jaw, low-set ears	--	n.r.
Barron-Diaz et al. [27]	Proband	25/F	VT	Limb weakness	n.r.	n.r.	PVC, VT	441	Short stature, broad forehead, jaw hypoplasia, dental alterations, camptodactyly, clinodactyly, feet syndactyly, dental alterations	--	n.r.
	Sister	7/F	VT, weakness, syncopal episodes	Limb weakness	n.r.	n.r.	PVC, VT, arrhythmias, mitral regurgitation	**544**	Triangular face, scoliosis, road forehead, ptosis palpebral, mandibular hypoplasia, dental alterations, camptodactyly in hands, clinodactyly, feet syndactyly	--	n.r.
**Our Family** **Case**	**Uncle**	**34/M**	**Cramps, myalgias, muscle weakness**	**General muscle** **hypertrophy**	**4.2**	** **297** **	**Coronary artery by-pass graftings; IHD; sVT; sEVB; EVB**	**360**	**---**	**Metabolic syndrome**	**66**
	**Father**	**6/M**	**Cramps, myalgias post-exercise**	**Lower limbs**	**5.4**	**827**	**AMI; eccentric hypertrophy of LV; ICD implant sVT; nsVT**	**450**	**Dental crowding**	**Metabolic syndrome;** **OSA**	**68**
	**Cousin**	**24/F**	**Cramps;** **muscle weakness; myotonia**	**Lower limbs**	**3.9**	**171**	**Normal**	**370**	**Small ears**		**5**
	**Older brother**	**12/M**	**Cramps; muscle stiffness at lower limbs**	**Lower limbs**	**4.7**	**150**	**Sinus arrhythmia; decreased amplitude of T waves in LPL**	**380**	**Jaw hypoplasia; dental crowding; clinodactyly fifth toe; syndactyly of II/III fingers of hands**	**Obesity**	**39**
	**Proband**	**16/F**	**Premenstrual episodes of PP, muscle stiffness**	**Upper and lower limbs**	**4.4**	**97**	**Sinus Bradycardia; EVB during FU**	**370**	**Micrognathia, elusive chin, low set of ears, dental crowding, hypotelorism, mall hands/ feet**	**___**	**37**
	**Young brother**	**11/M**	**Muscle** **stiffness**	**Upper limbs**	**4.9**	** **426** **	**Sinus Bradycardia; transient WPM**	**370**	**Scoliosis, micrognathia, small ears, hypertelorism, short neck, fingers clinodactyly and syndactyly**	**Obesity**	**32**
**(b)**											
**ATS Sporadic Cases**											
Haruna et al. [24]	Proband	6/M	Muscle weakness	PP	n.r.	n.r.	PVC	339	Dysmorphic features	--	n.r.
Takahashi et al. [28]	Proband	5/F	Syncopal events, muscle weakness	Muscle weakness	Normal	n.r.	Long QT, prominent U wave, PVC, intractable VA	**490**	--	--	18
Subbiah et al. [29]	Proband	17/F	Muscle weakness, bVT	PP, episodes of weakness	n.r.	n.r.	Symptomatic EVB, bVT, prominent U wave	460	--	--	n.r.
Rajakulendran et al. [30]	Proband	9/F	Attacks of muscle weakness	Muscle weakness	Normal	n.r.	--	365	Short stature, micromelia, low set ears, clinodactyly of hands, syndactyly of toes	--	n.r.
Kimura et al. [31]	Proband	6/F	Syncopal events	PP	n.r.	n.r.	PVC	**508**	--	--	n.r.
	Proband	11/F	--	--	n.r.	n.r.	pVT	n.r.	Dysmorphic features	--	n.r.
	Proband	6/M	--	PP	n.r.	n.r.	bVT	**468**	Dysmorphic features	--	n.r.
	Proband	19/F	Syncopal events	--	n.r.	n.r.	bVT	400	Dysmorphic features	--	n.r.
	Proband	12/M	--	--	n.r.	n.r.	bVT, pVT, PVC	427	Dysmorphic features	--	n.r.
	Proband	6/F	--	--	n.r.	n.r.	pVT, PVC	392	--	--	n.r.
	Proband	5/M	--	--	n.r.	n.r.	pVT, PVC	Normal	--	--	n.r.
Lefter et al. [32]	Proband	10/M	Lower limb weakness	PP, myalgia, weakness, fixed proximal myopathy	Normal	**850**	Prominent U wave	n.r.	Short stature, micromelia, micrognatia, low set ears, fifth digit clinodactyly, syndactyly of the left foot digits 2–3	--	19
Jung et al. [33]	Proband	6/M	pVT	Paralysis of lower limbs	**2.5**	n.r.	pVT	n.r.	Micrognatia, clinodactyly	--	25
Luo et al. [34]	Proband	11/M	PP	PP	Normal	n.r.	--	n.r.	--	--	n.r.
Horigome et al. [35]	Proband	6/M	PP	PP	n.r.	n.r.	bVT	n.r.	Short stature, dysmorphic features	--	n.r.
	Proband	19/M	PP	PP	n.r.	n.r.	PVC	n.r.	Dysmorphic features	--	n.r.
	Proband	24/F	PP	PP	n.r.	n.r.	PVC	n.r.	Short stature, dysmorphic features	--	n.r.
	Proband	28/F	PP	PP	n.r.	n.r.	bVT	n.r.	Short stature, dysmorphic features	--	n.r.
	Proband	54/F	PP	PP	n.r.	n.r.	PVC	n.r.	Dysmorphic features	--	n.r.
Yang et al. [36]	Proband	7/M	Syncopal events	Lower limb myotonia	**3.9**	n.r.	PVC, bVT, prominent U wave	420	Short stature, mandibular hypoplasia, single palmar crease, long bone, over hyper-extension	--	n.r.

Legenda. “--“: absence of signs indicated in the column. AMI: acute myocardial infarction; bVT: bidirectional ventricular tachycardia; CK: creatine kinase; CM: cardiomyopathy; DCM: dilated cardiomyopathy; EVB: ectopic ventricular beats; ICD: implantable cardioverter defibrillator; IHD: ischemic heart disease; LPL: left precordial leads; LQTS: long QT syndrome; LV: left ventricle; nsVT: not sustained ventricular tachycardia; OSA: obstructive sleep apnea; PP: periodic paralysis; pVT: polymorphic ventricular tachycardia; QTc: corrected QT interval in milliseconds; sVT: supra ventricular tachycardia; VA: ventricular arrhythmia; VF: ventricular fibrillation; VT: ventricular tachycardia; WPM: wandering pacemaker; n.r.: not reported. Metabolic syndrome includes hypertension, obesity, type 2 diabetes, and dyslipidemia; FU: follow-up. In bold, our family case; in red are abnormal values.

## Data Availability

Due to privacy, data is available upon request, subject to evaluation by the corresponding author.
